# Do patients benefit from omega-3 fatty acids?

**DOI:** 10.1093/cvr/cvad188

**Published:** 2024-01-22

**Authors:** Samuel C R Sherratt, R Preston Mason, Peter Libby, Ph Gabriel Steg, Deepak L Bhatt

**Affiliations:** Department of Molecular, Cellular, and Biomedical Sciences, University of New Hampshire, Durham, NH, USA; Elucida Research LLC, Beverly, MA, USA; Elucida Research LLC, Beverly, MA, USA; Department of Medicine, Cardiovascular Division, Brigham and Women’s Hospital, Harvard Medical School, Boston, MA, USA; Department of Medicine, Cardiovascular Division, Brigham and Women’s Hospital, Harvard Medical School, Boston, MA, USA; Université Paris-Cité, INSERM_UMR1148/LVTS, FACT (French Alliance for Cardiovascular Trials), Assistance Publique–Hôpitaux de Paris, Hôpital Bichat, Paris, France; Mount Sinai Fuster Heart Hospital, Icahn School of Medicine at Mount Sinai, 1 Gustave L. Levy Place, NewYork 10029-5674, NY, USA

**Keywords:** Omega-3 fatty acids, Eicosapentaenoic acid, Docosahexaenoic acid, Cardiovascular outcome trials, Atherosclerosis, Endothelial function, Cholesterol, Lipid oxidation

## Abstract

Omega-3 fatty acids (O3FAs) possess beneficial properties for cardiovascular (CV) health and elevated O3FA levels are associated with lower incident risk for CV disease (CVD.) Yet, treatment of at-risk patients with various O3FA formulations has produced disparate results in large, well-controlled and well-conducted clinical trials. Prescription formulations and fish oil supplements containing low-dose mixtures of eicosapentaenoic acid (EPA) and docosahexaenoic acid (DHA) have routinely failed to prevent CV events in primary and secondary prevention settings when added to contemporary care, as shown most recently in the STRENGTH and OMEMI trials. However, as observed in JELIS, REDUCE-IT, and RESPECT-EPA, EPA-only formulations significantly reduce CVD events in high-risk patients. The CV mechanism of action of EPA, while certainly multifaceted, does not depend solely on reductions of circulating lipids, including triglycerides (TG) and LDL, and event reduction appears related to achieved EPA levels suggesting that the particular chemical and biological properties of EPA, as compared to DHA and other O3FAs, may contribute to its distinct clinical efficacy. *In vitro* and *in vivo* studies have shown different effects of EPA compared with DHA alone or EPA/DHA combination treatments, on atherosclerotic plaque morphology, LDL and membrane oxidation, cholesterol distribution, membrane lipid dynamics, glucose homeostasis, endothelial function, and downstream lipid metabolite function. These findings indicate that prescription-grade, EPA-only formulations provide greater benefit than other O3FAs formulations tested. This review summarizes the clinical findings associated with various O3FA formulations, their efficacy in treating CV disease, and their underlying mechanisms of action.

**This article is part of the Spotlight Issue on Obesity, Metabolism, and Diabetes.**

## 1. Introduction to omega-3 fatty acids

**Figure 1:**
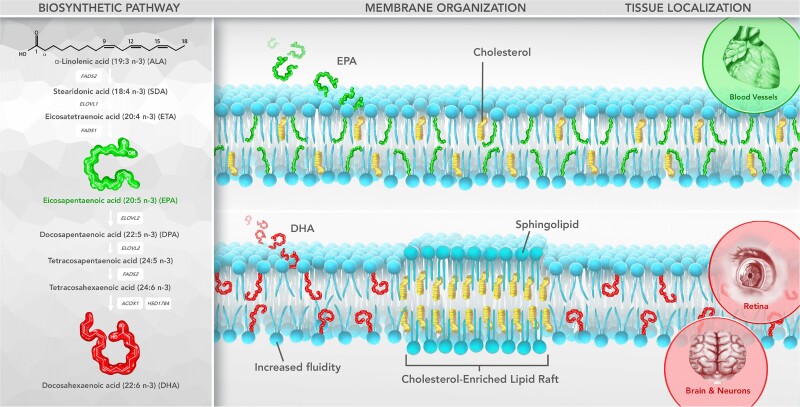
EPA and DHA can be synthesized from ALA, and adopt distinct orientations in the lipid bilayer. Production of EPA and DHA from ALA is an inefficient process, thus the most effective route to obtaining these O3FAs is often through the diet or pharmaceutical formulations. Within the membrane, EPA and DHA adopt distinct orientations and have contrasting effects on membrane fluidity. EPA has an extended, stable conformation and maintains normal cholesterol distribution and overall membrane fluidity, while DHA rapidly isomerizes on a nanosecond time scale, increases fluidity, and displace cholesterol into distinct domains, often rich in sphingolipids. DHA is known to often concentrate in retina and neuronal cell membranes, while EPA may concentrate in the membranes of endothelial cells and other vascular cells. FADS1, Δ^5^-desaturase; FADS2, Δ^6^-desaturase; ELOVL1, elongase of very long chain fatty acids protein 1; ELOVL2, elongase of very long chain fatty acids protein 2; ACOX1, peroxisomal acyl-coenzyme A oxidase 1; HSD1784, peroxisomal multifunctional enzyme type 2.

Omega-3 fatty acids (O3FAs) abound in nature, most notably in marine oily fish and plant-based sources including flaxseed oil, chia seeds, echium seeds, and walnuts.^1,2^ As humans lack the enzymes responsible for adding the ω-3 double bond at the methyl ends of fatty acids (the end opposite the carboxylic acid moiety, see *Figure 1*), O3FAs are considered essential fatty acids and must be acquired through the diet.^2^ Long chain O3FAs, including eicosapentaenoic acid (EPA, 20:5 n-3) and docosahexaenoic acid (DHA, 22:6 n-3), can be derived from α-linolenic acid (ALA, 18:3 n-3) through a series of elongase and desaturase reactions (*Figure 1*). A parallel pathway can synthesize omega-6 fatty acids (O6FA) (including arachidonic acid or AA, 20:4 n-6), which starts from linoleic acid (18:2 n-6) acquired from dietary sources and shares many key elongase and desaturase enzymes.^1,3^

Although humans can generate O3FAs such as EPA and DHA from ALA obtained through the diet, this process is inefficient, particularly in men compared with pre-menopausal women.^4^ Studies have shown that ≤8% of ALA was converted into EPA (and DHA in subsequent metabolic steps) in men while approximately 30% of ALA was converted to EPA and other metabolites in women of reproductive age.^5–8^ This sex-specific effect results from elevated levels of estrogens in pre-menopausal women, which increase conversion of ALA to DHA in likely support of foetal development.^8,9^ Because of the low conversion, the most efficient way to increase these essential fatty acids is through the consumption of fish or prescription-grade formulations, especially in patients at risk for cardiovascular disease (CVD) as increased O3FA levels link to reduced risk of CV mortality.

Before the widespread use of statins, numerous observational studies found beneficial associations between increased O3FA intake (mostly through diet) and decreased CVD risk in various patient populations (see the review by Breslow for a comprehensive summary of these studies).^10^ A 2004 meta-analysis of 13 cohorts from 11 observational studies, spanning from 1963 to 2003, found a pooled, relative risk reduction in coronary heart disease (CHD) of 15% with fish consumption.^11^ These promising results extended to randomized controlled trials (RCTs) as well.

The Diet and Reinfarction Trial (DART) showed a 29% relative risk reduction in all-cause mortality in men with previous myocardial infarction (MI) randomized to a diet of fatty fish.^12^ In contrast to these positive findings, other studies reported little to no CV benefit with increased O3FA intake;^13–18^ and still others found benefit with dietary fish intake but not with supplementation.^19–21^ In the most recent Cochrane systemic review and meta-analysis of ‘Omega-3 fatty acids for the primary and secondary prevention of cardiovascular disease,’ the authors pooled 86 RCTs of O3FA intake and found no high-certainty evidence for CV benefit in primary or secondary prevention and only slight reductions in CHD mortality risk in low-certainty evidence with a number needed to treat (NNT) of 334.^15^ Thus, the evidence for CV benefit with O3FAs from supplementation or dietary increase is mixed, perhaps as a result of heterogeneity among supplements, diets, and entry criteria, potentially obfuscating clinical outcomes.

## 2. Fish oil supplements vs. prescription formulations

The widespread use of fish oil dietary supplements (FODS) greatly increases access to O3FAs for patients with aversions to fish consumption. Often, FODS contain low levels (<1 g) of EPA and DHA mixed with other oils harvested from source fish. However, these products do not undergo rigorous oversight by the Food and Drug Administration (FDA) or other global regulatory agencies, permitting product-to-product and batch-to-batch variations in fatty acid content, potential exposure to oxidized constituents, and other issues that raise concern about their chemical integrity.^22^ Analysis of capsule contents frequently shows elevated levels of saturated fats, primary (peroxide) and secondary (aldehyde) lipid oxidation products, and deviations from the labelled amounts of EPA and DHA.^22–27^ While inconsistent levels of O3FAs may compromise any intended benefit of these supplements, the presence of saturated fats, excess calories, and oxidized oils might actually cause harm, especially if consumed in large quantities and for long periods of time. Animal studies have shown that ingestion of oxidized lipid increases aortic atheroma size, plasma LDL levels, and lipid oxidation products found in circulating lipoproteins as well as in the liver.^28–32^ By contrast, the stringent requirements for prescription O3FA formulations mandated by the FDA result in products with greater purity, negligible lipid oxidation by-products, and reproducible O3FA content.^23,25^ For these reasons, FODS are not suitable substitutes for prescription O3FA formulations for CV patients.

Encouraging early clinical trials using O3FA supplements for secondary prevention of CVD events inspired further investigation into this field (for a detailed summary of CV outcome trials using O3FA formulations, see *Table 1*). Specifically, the Gruppo Italiano per lo Studio della Sopravvivenza nell’Infarto Miocardico—Prevenzione (GISSI-P) trial published in 1999 showed that in patients with a recent (≤3 months) MI, administration of 1 g/day O3FA supplement significantly reduced the primary endpoint of composite major adverse cardiovascular events (MACE).^33^ This was one of the first randomized O3FA supplement trials to show clinically significant benefit for patients, especially against the backdrop of trials based on dietary/lifestyle interventions, which are very difficult to interpret given the inherent challenges of residual confounding.

**Table 1:** Summary of CVOTs using O3FA formulations

Trial (year published)	No. of patients randomized	Inclusion criteria	O3FA formulation	Therapeutic dose	Per Capsule content	Statin background	Primary endpoint	Primary endpoint HR (95% CI), *P*-value
GISSI-P^a^ (1999)	11 324	Patients w/ recent MI (≤3 months)	EPA + DHA EE	1 g/day	∼866 mg EPA + DHA (1:2 EPA:DHA ratio)	5% at baseline, 46% by EOS	All-cause death, non-fatal MI, non-fatal stroke	0.85 (0.74–0.98) *P* = 0.023
JELIS^a^ (2007)	18 645	Hypercholesterolaemic, LDL-C ≥ 170 mg/dL	IPE	1.8 g/day	≥294 mg IPE	100%	Any major coronary event	0.81 (0.69–0.95) *P* = 0.011
GISSI-HF^b^ (2008)	6975	HF patients ≥ 18 years w/ NYHA Class II–IV	EPA + DHA EE	1 g/day	∼866 mg EPA + DHA (1:2 EPA:DHA ratio)	23%	(1) Time to death *and* (2) time to death or hospitalization for CV reasons	(1) 0.91 (0.83–0.99) *P* = 0.041 (2) 0.90 (0.81–0.99) *P* = 0.045
ASCEND (2018)	15 341	Patients ≥ 40 years w/ DM but no CVD evidence	EPA + DHA EE	1 g/day	460 mg EPA + 380 mg DHA	∼75%	Non-fatal MI, non-fatal stroke, TIA, vascular death	0.97 (0.87–1.08) *P* = 0.55
VITAL^b,c^ (2019)	25 871	Men ≥ 50 years and women ≥ 55 years, no CVD evidence	EPA + DHA EE	1 g/day	460 mg EPA + 380 mg DHA	∼35%	(1) MI, stroke, or CV death *and* (2) invasive cancer	(1) 0.92 (0.8–1.06) *P* = 0.24 (2) 1.03 (0.93–1.13) *P* = 0.56
REDUCE-IT (2019)	8179	TG levels 150–499 mg/dL w/ ASCVD or diabetes w/ ≥1 other risk factors	IPE	4 g/day	≥960 mg IPE	100%	CV death, non-fatal MI, non-fatal stroke, coronary revasc, or unstable angina	0.75 (0.68–0.83) *P* < 0.001
STRENGTH (2020)	13 078	TG levels 180–499 mg/dL and HDL-C ≤ 40 mg/dL w/ ASCVD or diabetes w/ ≥1 other risk factors	EPA + DHA FFA	4 g/day	≥750 mg EPA + DHA	100%	CV death, non-fatal MI, non-fatal stroke, coronary revasc, or unstable angina	0.99 (0.90–1.09) *P* = 0.84
OMEMI (2021)	1027	Elderly patients (70–82 years) w/ recent AMI	EPA + DHA FFA	1.8 g/day	310 mg EPA + 220 mg DHA	96%	AMI, revasc, stroke, all-cause death, or HF hospitalization	1.08 (0.82–1.41) *P* = 0.60
RESPECT-EPA^a^ (2022)	2506	EPA/AA ratio < 0.4 Chronic CAD ≥ 1-month prior statin use	IPE	1.8 g/day	≥294 mg IPE	100%	CV death, non-fatal MI, non-fatal stroke, revasc, unstable angina	0.79 (0.62–1.00) *P* = 0.055

AA, arachidonic acid; AMI, acute myocardial infarction; ASCVD, atherosclerotic cardiovascular disease; CI, confidence interval; CAD, coronary artery disease; DHA, docosahexaenoic acid; DM, diabetes mellitus; EE, ethyl ester; EOS, end-of-study; EPA, eicosapentaenoic acid; FFA, free fatty acid; HDL-C, high density lipoprotein cholesterol; HF, heart failure; HR, hazard ratio; IPE, icosapent ethyl; LDL-C, low density lipoprotein cholesterol; MI, myocardial infarction; NYHA, New York Heart Association; Revasc, revascularization; TG, triglyceride; TIA, transient ischaemic attack.

^a^Denotes trials that were open label.

^b^GISSI-HF and VITAL pre-specified co-primary endpoints, thus we report the HR and *P*-values for each.

^c^In VITAL, patients randomized to the active treatment arm were also given 2000 IU/day vitamin D3.

The subsequent GISSI-Heart Failure (GISSI-HF) trial showed more promising benefits of O3FA supplementation.^34^ Here, investigators found that treatment with the same 1 g/day O3FA supplement in patients with established heart failure (New York Heart Association class II–IV) with varying left ventricular function reduced death from any cause by 9% and death from cardiovascular events by 10%. Together, these trials generated enthusiasm for O3FA intervention for patients with established CVD; however, both trials lack relevance as compared with more recent trials of O3FA formulations due to substantial changes in standard of care. Baseline statin use in GISSI-P was 5% initially and rose to 46% by end of study; baseline statin use in GISSI-HF was 23%.^35^ As a result, low-dose, mixed EPA/DHA O3FA supplementation for secondary prevention in patients with established CVD cannot be compared to contemporary care, which includes high-intensity statin therapy. The meta-analysis by Khan and colleagues supports this perspective. They investigated the effects of EPA monotherapy as compared with EPA/DHA mixed formulations across 36 RCTs—including GISSI-P and GISSI-HF—in nearly 150 000 patients.^36^ The investigators found that either EPA monotherapy or EPA/DHA mixed formulation therapy reduced CV mortality (rate ratio, RR = 0.82 (0.68–0.99) and 0.94 (0.89–0.99), respectively; both *P* < 0.05) and CHD events (RR = 0.73 (0.62–0.85) and 0.94 (0.89–0.99), respectively; both *P* < 0.05). While EPA monotherapy exhibited superiority to EPA/DHA mixed formulations, both therapies showed significant benefits for patients. However, *post hoc* analysis of only contemporary care trials of EPA/DHA mixed formulations, which excluded earlier trials like GISSI-P and GISSI-HF where statin background therapy was lower, showed no significant reductions in CV mortality and CHD events (RR = 0.96 (0.90–1.03) and 0.95 (0.90–1.00), respectively; both *P* > 0.05).^37^

Aung *et al*.^38^ reported similar findings in a 2018 meta-analysis. This compilation amassed data from nearly 78 000 patients across ten RCTs, nine of which employed supplements containing various levels of EPA and DHA and one (the Japan EPA Lipid Intervention Study, JELIS) used a prescription strength EPA-only formulation. Total EPA + DHA content also varied across the included trials, with six of the trials using ≤1 g EPA + DHA and only one of the remaining trials used >2 g EPA + DHA. Statin use across the studies ranged from 23% (GISSI-HF) to 100% (JELIS), and each trial included patients at high risk for CVD. The results showed that O3FA supplementation did not reduce fatal and non-fatal CHD, stroke, revascularization events, or any major vascular event (RR =0.96 (0.90–1.01), 1.03 (0.93–1.13), 0.99 (0.94–1.04), and 0.97 (0.93–1.01), respectively; all *P* > 0.05). Despite the necessary caveats mentioned above (heterogeneity of O3FA dose, standard of care, trial design, and etc.), this meta-analysis offers a clearer interpretation of the clinical value of O3FA treatment beyond lifestyle changes; namely, the bulk of the evidence shows that the administration of low-dose (<2 g/day) mixed EPA/DHA supplements does not significantly reduce CVD events. Following its publication, two more large RCTs of low-dose (1 g/day) O3FA supplements for primary prevention of CVD reported.^39,40^ The Vitamin D and Omega-3 Trial (VITAL) and A Study of Cardiovascular Events in Diabetes (ASCEND) enrolled large cohorts (25 871 and 15,480, respectively) without established CVD, and neither showed a significant reduction in CVD events as compared with placebo. Taken together, low-dose and mixed O3FA formulations do not appear to significantly attenuate primary and secondary CVD events.

## 3. EPA monotherapy vs. EPA/DHA combination therapy: JELIS, REDUCE-IT, STRENGTH, and RESPECT-EPA

Despite the shortcomings of low-dose, mixed EPA/DHA formulations in patients with or without established CVD, clinical trials of higher dose EPA monotherapy have demonstrated benefit. The above-mentioned JELIS trial from 2007 enrolled 18 645 primary or secondary prevention participants and showed a relative risk reduction (RRR) of 19% (*P* = 0.011) for a composite of major coronary events in patients with hypercholesterolaemia when 1.8 g/day icosapent ethyl (IPE; a pure, ethyl ester form of EPA) was added to a statin regimen compared with statin treatment alone.^41^ It is noteworthy that JELIS was a blinded end point, open-label trial with no placebo, and was conducted in Japanese subjects who at the time likely had greater fish consumption than Western populations, and had correspondingly higher baseline EPA concentrations in blood. Among the components of the primary endpoint, significant reductions were observed in hospitalization for unstable angina (24%) and non-fatal coronary events (19%). Interestingly, although there was no pre-specified inclusion criteria based on triglyceride (TG) level, the average baseline TG levels were near normal (153 mg/dL), and the overall TG reduction was modest (9%), a *post hoc* analysis found that patients with TG levels above 150 mg/dL combined with low high density lipoprotein cholesterol (HDL-C, <40 mg/dL) experienced a 53% reduction in major coronary events (*P* = 0.043).^42^

Subsequent trials using 4 g/day EPA ethyl ester in patients with severe (≥500 and ≤2000 mg/dL; the MARINE trial) and persistent (≥200 and <500 mg/dL; the ANCHOR trial) hypertriglyceridaemia revealed consistent reductions in median TG levels compared with baseline (27% reduction and 18% reduction, respectively), which correlated with increased plasma EPA levels.^43–45^ As the ANCHOR trial also included patients treated with statins, the potential benefit of lowering TG levels in this population, combined with the promising results from JELIS, warranted further investigation into the effects of IPE on CVD outcomes, as subsequently investigated in the Reduction of Cardiovascular Events with Icosapent Ethyl–Intervention Trial (REDUCE-IT).

REDUCE-IT tested the effects of IPE on residual CVD risk in patients with statin-stabilized LDL-C.^46^ This multicentre, placebo-controlled trial randomized 8179 patients with residually high TGs (≥135 and <499 mg/dL) and established CVD or diabetes with at least one additional risk factor to 4 g/day IPE or placebo. Approximately 71% of the patients were enrolled based on secondary prevention, and the remaining 29% were enrolled for primary prevention with diagnosed diabetes and at least one other risk factor. There was a RRR of 25% (HR = 0.75 (0.68–0.83), *P* < 0.001) and an absolute risk reduction of 4.8% (3.1–6.5) associated with the primary endpoint, which was five component composite major adverse cardiovascular events (MACE, including CV death, non-fatal MI, non-fatal stroke, hospitalization for unstable angina, or coronary revascularization). The number needed to treat in 5 years to prevent one event (NNT) was 21 for the primary endpoint.

At the first pre-specified interim analysis, after approximately 60% expected events had occurred, the Data Safety Monitoring Board detected a risk reduction of the primary endpoint with IPE (HR: 0.77 (0.68–0.87), *P* < 0.001) and that this had reached significance after approximately 21 months following randomization, indicating an early benefit with IPE treatment.^47^ Pre-specified hierarchical analysis of endpoints showed that IPE treatment lowered risk of fatal or non-fatal MI by 31% (HR: 0.69 (0.58–0.81), *P* < 0.001), fatal or non-fatal stroke by 28% (HR: 0.72 (0.55–0.93), *P =* 0.01), and CV death by 20% (HR: 0.80 (0.66–0.98), *P =* 0.03). Total (first and subsequent) ischaemic events fell by 30% (RR: 0.70 (0.62–0.78), *P* < 0.001) in the IPE treatment arm, and first coronary revascularizations were reduced by 34% (HR: 0.66 (0.58–0.76), *P* < 0.001).^48,49^

*Post hoc* and pre-specified subgroup analyses revealed remarkable consistency of benefit with IPE among patients with various CVD histories. The treatment reduced risk of the primary endpoint in patients with prior MI (26%, HR: 0.74 (0.65–0.85), *P* < 0.001), prior percutaneous coronary intervention (PCI, 34%, HR: 0.66 (0.58–0.76), *P* < 0.001), prior coronary artery bypass graft (CABG, 24%, HR: 0.76 (0.63–0.92), *P* = 0.004), and across a range of cigarette smoking history (23%, HR: 0.77 (0.68–0.87), *P* < 0.0001).^50–53^ In the 3146 patients enrolled in the USA, who historically have experienced attenuated benefits compared with patients enrolled in CVOTs from other countries, had a 31% relative risk reduction in the primary composite endpoint (HR: 0.69 (0.59–0.80), *P* < 0.001).^54^ The risk reduction for patients was consistent regardless of statin type, including lipophilic statins such as atorvastatin (HR: 0.79 (0.67–0.93), *P =* 0.006) or more hydrophilic statins such as rosuvastatin (HR 0.73 (0.57–0.94), *P* = 0.01).^55^ Risk fell consistently in patients with pre-specified categories of estimated glomerular filtration rate (eGFR); <60 mL/min/1.73m^2^ (29%, HR: 0.71 (0.59–0.85), *P* < 0.001), ≥60 and <90 mL/min/1.73m^2^ (20%, HR: 0.80 (0.70–0.92), *P* = 0.001), or ≥90 mL/min/1.73m^2^ (30%, HR: 0.70 (0.56–0.89), *P* = 0.003).^56^ Together with the earlier results from JELIS, the REDUCE-IT results show a substantial, highly-significant reduction in CVD risk in statin-treated patients with IPE. Both JELIS and REDUCE-IT, showed an inverse relationship between achieved plasma/serum EPA levels and event rate, a further indication that EPA itself contributes to the cardiovascular benefits of IPE treatment.^57,58^

The newer Randomized trial for Evaluation in Secondary Prevention Efficacy of Combination Therapy Statin and Eicosapentaenoic Acid (RESPECT-EPA) trial provided further information regarding CVD risk reduction with IPE.^59^ This open-label trial from Japan followed a similar design as JELIS with some key differences in the inclusion criteria. As in JELIS, statin-treated patients randomly received 1.8 g/day IPE or continued statin therapy alone. Patients in this trial also had documented coronary artery disease (CAD). Additionally, patients were screened for their baseline plasma EPA/AA ratio, and patients with an EPA/AA ratio < 0.4 were included in the randomization. The primary endpoint was a composite of CV death, non-fatal MI, non-fatal stroke, unstable angina requiring emergency hospitalization and subsequent coronary revascularization, or revascularization based on other clinical findings. Although treatment with IPE indicated a potential reduction in the primary endpoint, this did not reach statistical significance (*Table 1*). Of note, this trial was likely underpowered as the actual cumulative event rate in the IPE arm (10%) was less than estimated at the outset of the trial (12%).

An interesting *post hoc* analysis of RESPECT-EPA evaluated the effects of IPE on EPA/AA ratio on the primary endpoint. Patients in the IPE arm who did not achieve a large increase in plasma EPA levels from baseline (<30 µg/mL) and patients in the control arm who did achieve a large increase in EPA levels from baseline (>30 µg/mL) were excluded. Evaluation of the primary endpoint in the remaining patients showed a statistically significant reduction with IPE treatment (HR: 0.73 (0.55–0.95), *P* = 0.020). As with all *post hoc* analyses of clinical trials, especially those in which the pre-specified intention-to-treat analysis did not meet statistical significance, this result can only serve to generate more hypotheses. Further research is warranted to investigate the use of IPE in patients with established atherosclerotic cardiovascular disease (ASCVD) and low EPA/AA ratio.

Recent CVOTs with EPA/DHA mixed formulations, in combination with statin therapy, have not shown the same benefit as EPA monotherapy. Approximately one year after the REDUCE-IT trial was published, the Long-Term Outcomes Study to Assess Statin Residual Risk with Epanova in High Cardiovascular Risk Patients with Hypertriglyceridemia (STRENGTH) trial was halted early for futility.^60^ This trial enrolled a patient population similar to that in REDUCE-IT and administered mixed EPA/DHA carboxylic acids (4 g/day) on top of statin therapy. Although there was a similar fall in TG levels (19%) as observed in REDUCE-IT, there was no reduction of the same primary endpoint (5-point MACE). Subsequent analysis of STRENGTH showed that the lack of benefit was consistent across all achieved EPA levels.^61^ However, because EPA and DHA were co-administered, it is impossible to separate the increases in EPA from concomitant and significant increases in DHA in the active treatment arm. This point raises a potential counter-regulatory action of DHA in the context of atherosclerosis and CVD.^62,63^ After STRENGTH, the OMega-3 fatty acids in Elderly with Myocardial Infarction (OMEMI) trial, which also used a mixed EPA/DHA formulation (1.8 g/day), failed to reduce CVD events in older patients with a recent acute MI.^64^ Statin adherence in the STRENGTH and OMEMI trials were 100 and 96%, respectively, thus differentiating them from previous trials of mixed EPA/DHA formulations that had shown significant CVD risk reduction before the era of widespread statin use (e.g. GISSI-P and GISSI-HF).

## 4. Mineral oil: inert placebo or active comparator?

Concerns have been raised about the placebo (mineral oil) used in REDUCE-IT, leading to the suggestion that some portion of the large benefit observed with IPE was erroneously attributed to IPE and was, in fact, caused by detrimental effects of mineral oil. By comparison, the STRENGTH trial used a corn oil placebo. A *post hoc* analysis of REDUCE-IT showed that certain well-established inflammatory and lipid biomarkers, including hsCRP, IL-1β, and LDL-C, increased significantly in the placebo arm from baseline while IPE had minimal effect on these markers despite similar baseline levels.^65^ Thus, the event reduction with IPE was not associated with any of these biomarkers. These data are sometimes cited to support theoretically negative effects of mineral oil and cast doubt on the magnitude of clinical benefits of IPE.

These biomarker changes, while statistically significant, were very small on an absolute scale and often below the lower detection limit of the assays, raising the question of their clinical significance. For instance, levels of hsCRP increased from 2.1 to 2.8 mg/L after 12 months in the placebo arm. By contrast, baseline levels of hsCRP in the JUPITER trial were 4.3 mg/L, more than 1.5 times greater than the *maximum* levels in the placebo arm in REDUCE-IT.^66^ Similarly, levels of IL-1β increased from 0.06 to 0.09 pg/mL at the last visit, which is more than 16-fold less than the baseline levels in the CIRT trial.^67^ Together, these data show that the REDUCE-IT population differed markedly from patients in other atherosclerotic CVD trials with regard to inflammatory status, so it may not be surprising that event reduction with IPE did not correlate with the IL-1β-IL-6-hsCRP axis.^68^ Furthermore, it is also possible that these differences in biomarkers between arms might have been due to icosapent ethyl blunting a rise in biomarkers that would have otherwise occurred, and that the small increases in the placebo arm were part of the natural history of the underlying risk profile of this specific patient population. A recent comparison of two imaging trials that used mineral oil (EVAPORATE) or a predominantly cellulose placebo (Garlic-5) showed no significant difference in total and non-calcified plaque volume as measured by coronary computed tomographic angiography (CTA).^69^

Pharmaceutical grade mineral oil, as used in placebo-controlled trials, contains saturated, aliphatic hydrocarbons ranging in length from approximately C15-C26.^70^ Previous studies have shown that saturated fatty acids (18:0 and 20:0) do not alter lipoprotein oxidation, membrane oxidation, or cholesterol crystal domain formation.^71^ Historically, there has been no reproducible or clinically-relevant pattern of lipid or inflammatory biomarker change with mineral oil.^70^ The Food and Drug Administration (FDA) conducted an exploratory, *post hoc* analysis of these data to estimate what effect, if any, the use of a mineral oil comparator could have on the overall reduction in primary endpoint.^70,72^ They concluded that even if the increase in hsCRP and LDL-C between the treatment arms was entirely attributable to the mineral oil placebo, this would not account for more than 3% of the 25% risk reduction with IPE treatment. A separate *post hoc* analysis evaluated the primary endpoint event rate in both arms among patients with and without increases in hsCRP or LDL.^70^ The event rate did not differ between patients who did or did not experience an increase in hsCRP or LDL, and the event reduction with IPE was also consistent among these two groups of patients. These data further suggest that the changes in hsCRP and LDL do not account for the event reduction with IPE in REDUCE-IT.

Some have hypothesized that the increase in LDL-C levels could result from decreased statin absorption with mineral oil use. However, a recent animal study showed no change in the absorption or bioavailability of lipophilic and hydrophilic statins when co-administered with mineral oil as compared with water.^73^ As well, in the study mentioned that examined IPE benefit as a function of baseline statin use in REDUCE-IT, there was no difference in the benefit with lipophilic or hydrophilic statin use. If mineral oil were interfering with statin absorption, one might expect a differential effect based on hydrophilic or lipophilic properties.^55^

Finally, an *in vitro* study compared directly the effects of pharmaceutical grade placebo oils (mineral and corn oil) to equimolar levels of EPA or DHA on rates of lipid oxidation in different sized ApoB-containing lipoproteins (small dense LDL, VLDL) as well as in models membranes.^74^ Oxidation of LDL favours foam cell formation during atherosclerosis, while membrane oxidation can promote cell injury and death.^75^ At a pharmacologic concentration, EPA had potent and sustained antioxidant effects in LDL and membranes compared with DHA as well as the placebo oils, which had no effect on lipid oxidation rates. The distinct antioxidant benefits of EPA may represent an important atheroprotective mechanism, independent of placebo oil selection. It therefore remains highly unlikely that any meaningful part of the benefits with IPE observed in REDUCE-IT resulted from deleterious effects of 2 cc twice a day of pharmaceutical grade mineral oil.

## 5. Role of TGs in CVD event reduction—is it a contributing mechanism?

Although TG levels fell significantly with IPE treatment (19.7%), the benefit with IPE treatment did not associate with baseline or changes in plasma TG levels or other well-established lipid and inflammatory biomarkers (e.g. hsCRP and LDL-C). Rather, serum EPA levels did predict risk reduction with IPE treatment.^58^ This observation suggests that although TG concentrations might identify patients who could benefit from add-on therapy to statins, modest reduction of TGs itself is unlikely to be mechanistic in prevention of CVD.^76^ The lack of risk reduction in STRENGTH despite a similar degree of TG lowering supports this hypothesis. Elevated TG levels do associate with increased CVD risk.^77^ Similar to the early O3FA supplementation RCTs, before statins were widely used, early clinical investigations using fibrates showed promising benefits with TG reduction with regard to CV events. The Veterans High-Density Lipoprotein Cholesterol Intervention Trial (VA-HIT) showed a RRR of 22% in the primary outcome of non-fatal MI and death from coronary causes and a 31% reduction in TGs with gemfibrozil in men with diagnosed CHD and low HDL (≤40 mg/dL).^78^ Preceding this trial, the Helsinki Heart Study (HHS) found a RRR of 34% for cardiac death and fatal or non-fatal MI and a 35% reduction in TGs with gemfibrozil in men with mixed dyslipidaemia enrolled for primary prevention.^79,80^

However, as statins became established as the standard of care regimen, evidence for the benefits attributed to fibrates waned, as evidenced in the Action to Control Cardiovascular Risk in Diabetes (ACCORD) trial that failed to show reduction in MACE with fenofibrate in high CV risk patients with diabetes despite a ∼25% reduction in TGs.^81^ The story was similar for trials using niacin in statin-treated patients—no reduction in CVD risk was observed with niacin despite reductions in TGs (eg, AIM-HIGH, HSP2-THRIVE).^82,83^ Importantly, these trials did not pre-specify TG thresholds in determining the inclusion criteria. Despite the overall failure of the trials, *post hoc* analysis of ACCORD revealed promising results in patients with elevated TGs and low HDL, and a separate imaging trial (FIRST) found a similar benefit with statin (atorvastatin) plus fenofibrate in reducing carotid intima-media thickness only in patients with TG levels ≥ 175 mg/dL.^84^

With this in mind, the Pemafibrate to Reduce Cardiovascular Outcomes by Reducing Triglycerides in Patients with Diabetes (PROMINENT) trial examined the effects of pemafibrate, a selective peroxisome proliferator-activated receptor alpha modulator (SPPARM-α), on major CVD events in patients with type 2 diabetes and hypertriglyceridaemia (200–499 mg/dL) and an HDL ≤ 40 mg/dL on stable statin therapy.^85^ Pemafibrate exhibits greater affinity for PPAR-α than other fibrates, and reduced TGs 40–50% compared with placebo in early phase trials. In a sense, this trial gave the fibrate class an excellent opportunity to show clinically significant benefits for patients receiving contemporary care. Despite the clinical evidence that these patients may benefit more from TG lowering, this trial halted prematurely for futility.^86^ Pemafibrate reduced TGs by 26% and, notably, increased ApoB levels 4.8% compared to placebo. Whether the lack of CVD risk reduction was due to the increase in ApoB or the diminished impact of TG lowering in general, current evidence shows that fibrates offer no benefit for major adverse cardiovascular event outcomes in high-risk, statin-treated patients. Pemafibrate may however have benefits for diabetic microvascular disease and hepatopathies, issues that merit further study.

Other therapeutic strategies are currently being investigated for CV risk reduction linked to TG lowering, including antisense oligonucleotides or monoclonal antibodies targeting angiopoietin-like 3 (ANGPTL3) and apolipoprotein CIII (ApoCIII).^77^ Both ANGPTL3 and ApoCIII inhibit lipoprotein lipase (LPL), thus increasing circulating TG rich lipoprotein (TGRL) levels. In the TRANSLATE-TIMI 70 trial, vupanorsen (an ANGPTL3-targeted antisense oligonucleotide) reduced TG levels by as much as 56% compared with placebo in patients with elevated TGs and non-HDL-C.^87^ Despite these effects, the sponsor discontinued clinical development of this therapy citing a lack of sufficient TG- and non-HDL-C-lowering coupled with increases in liver fat and other unwanted actions.^88^ The monoclonal antibody against ANGPTL3, evinacumab, has shown promising reductions in LDL-C in patients with homozygous familial hypercholesterolaemia and refractory hypercholesterolaemia.^89,90^ Targeting ApoCIII has also shown promising effects on lipid markers, as the antisense oligonucleotide volanesorsen significantly reduced TG by 77% compared to placebo in patients with familial chylomicronaemia syndrome.^91^ It remains to be seen whether this benefit will extend to patients with established CVD risk similar to the REDUCE-IT study population. Further investigation into the mechanistic contribution of ApoCIII reductions using antisense oligonucleotides will shed light on its impact in CVD. It appears that the benefit of IPE is largely independent of TG lowering, and the mechanism of action may directly involve effects of EPA and its various bioactive metabolites on various aspects of atherosclerotic cardiovascular disease pathophysiology (as summarized in *Table 2*).

**Table 2:** Comparison of EPA and DHA on various mechanisms of atherosclerosis and cardiovascular disease

*Clinical Findings*	*EPA*	*EPA+DHA*
Reduces Incidence of MACE in High-Risk, Statin-Treated Patients	+	−
***Mechanism of Action***		
Reduces plasma TG levels	+	+
Maintains normal membrane cholesterol distribution	+	−
Preserves membrane stability	+	−
Inhibits glucose-induced membrane cholesterol domains	+	−
Enhanced EC function/NO release and lipid antioxidant activity with statin	+	+/−
Reduces expression of adhesion molecules during inflammation	+	+
Inhibits lipoprotein oxidation	+	−
Serves as a source of specialized pro-resolving mediators	+	+
Localizes to vulnerable atherosclerotic plaque and increases plaque stability	+	−
Binds GPR120 to elicit anti-inflammatory actions	+	+
Improves glucose tolerance and insulin sensitivity	+	−

The ‘+’ indicates that there is evidence that this occurs, the ‘−’ indicates that there is evidence that this does *not* occur, ‘±’ indicates that there is evidence that this can occur sometimes, depending on the experimental conditions.

EC, endothelial cell; GPR120, G-protein coupled receptor 120; MACE, major adverse cardiovascular events; NO, nitric oxide; TG, triglyceride.

## 6. EPA mechanisms of action: insights from plaque morphology and imaging studies

Atherosclerotic plaque biology involves endothelial cell dysfunction, leucocyte activation, and lipid accumulation—all of which may link to CVD events through inflammatory mechanisms.^92^ Several recent reviews provide more complete discussions of atherosclerotic plaque initiation and progression.^92–95^ There is ongoing debate as to the overall contribution of ‘vulnerable’ plaques or plaques with thin fibrous caps to thrombotic events, and growing evidence points to inflammatory mediators (such as neutrophil extracellular traps, or NETs) brought on by endothelial erosion—the condition in which the endothelial monolayer sloughs and exposes the intima to leucocyte adherence and thrombus formation—as consistent contributors to ischaemic events.^92,96,97^

Conversely, favourable changes in plaque morphology, such as increasing fibrous cap thickness, reducing atheroma volume, and decreasing inflammatory cell concentrations within the plaque, would be expected to reduce the risk of ischaemic events.^96^ Animal studies of *ApoE*-deficient mice have shown that EPA, as compared to DHA, incorporates preferentially into plaques with thin fibrous caps following intake of these O3FAs in the diet.^98^ There was an inverse relationship between concentration of EPA and several of its signature metabolites—12-hydroxy-eicosapentaenoic acid (12-HEPE) and leukotriene B_5_ (LTB5)—in plaques and the thickness of the fibrous cap, and this correlated with larger decreases in intima thickness as compared with DHA-fed mice. Thus, EPA may provide particular localization and stabilizing effects on otherwise vulnerable plaques. These findings were supported by a study in humans who underwent carotid endarterectomy following 21-day administration of an EPA/DHA mixed formulation.^99^ EPA again preferentially incorporated into plaques compared to DHA, increased plaque stability and decreased plaque inflammation in proportion to its content.

Clinical imaging studies offer further insight into the beneficial effects of EPA on plaque morphology and may explain, at least in part, the differences observed in large CVOTs between EPA monotherapy and mixed EPA/DHA formulations. The EVAPORATE trial examined the effects of 4 g/day IPE on plaque progression, specifically low attenuation plaque (LAP) volume, using serial multidetector computed tomography (MDCT) in 80 patients matching the population evaluated in REDUCE-IT.^100^ Change in LAP was chosen as the primary endpoint as patients with a larger LAP volume (>4%) have increased risk for MI.^101^ After 18 months of treatment LAP volume fell by 17% compared with baseline in the IPE treatment arm while the placebo arm experienced a 109% increase compared with baseline (*P* = 0.006 between arms). Other characteristics of atherosclerotic plaques decreased with IPE as compared with baseline, specifically fibro-fatty, fibrous, total non-calcified, and total plaque fell by 34%, 20%, 19%, and 9%, as compared with placebo (all *P* < 0.01). Fractional flow reserve (FFR) measurements from the CT angiography data (FFR_CT_) on a cohort from EVAPORATE revealed early and significant improvement in coronary physiology and haemodynamics with IPE compared to placebo.^102^ As in REDUCE-IT, these changes in plaque morphology did not associate with changes in TGs or other lipid biomarkers.

The Combination Therapy of Eicosapentaenoic Acid and Pitavastatin for Coronary Plaque Regression Evaluated by Integrated Backscatter Intravascular Ultrasonography (CHERRY) trial performed in Japan used integrated backscatter intravascular ultrasound (IB-IVUS) to measure changes in coronary thin-cap fibroatheroma in patients with stable angina or acute coronary syndrome (ACS) who underwent successful PCI and were treated with EPA ethyl ester (IPE, 1.8 g/day) plus pitavastatin or pitavastatin alone.^103^ The combination of IPE and statin significantly decreased total atheroma volume and increased the EPA–AA ratio compared with statin treatment alone, indicating that EPA may facilitate plaque regression and stabilization through anti-inflammatory mechanisms that run counter to known pro-inflammatory effects of AA and its various pro-inflammatory metabolites, such as thromboxane A_2_ and leukotriene B_4_. Another trial from Japan using optical coherence tomography (OCT) found that the addition of IPE treatment (1.8 g/day) to statin therapy in patients with dyslipidaemia significantly increased fibrous cap thickness in concert with a decrease in macrophage accumulation, indicating improved indices of plaque stability.^104^

In contrast to these improvements with IPE, mixed EPA/DHA formulations have not shown significant changes in markers of plaque stability. The HEARTS trial investigated the effect of treatment with 3.4 g/day EPA/DHA ethyl esters on the primary endpoint of non-calcified plaque volume in patients with stable CAD.^105^ Statin adherence in this trial was 93–96% in the control (no O3FA treatments) group and treatment group. The study did not meet its primary endpoint in either intention-to-treat or per-protocol analyses.

## 7. EPA mechanisms of action: role of O3FA-generated lipid metabolites in atherosclerosis and inflammation

The central role that uncontrolled inflammation plays in atheroma initiation and disruption likely reflects an imbalance in its initiation *and resolution* signalling. Groundbreaking work by Serhan and colleagues has uncovered the effects of various lipid bioactive metabolites of O6FAs and O3FAs on restoring inflammatory homeostasis following acute injury.^106–110^ The precursor O3FA and O6FAs in the cell membrane are hydrolyzed from the phospholipid via phospholipase A2 (PLA2) to elicit various downstream cellular responses through their distinct bioactive lipid metabolites generated from intracellular cyclooxygenases (COXs), lipoxygenases (LOXs), and cytochrome P450 (CYP) enzymes.^111^ Under normal conditions, the complex web of eicosanoids derived from EPA and AA, along with specialized pro-resolving mediators (SPMs) from EPA, DHA, and DPA, is tightly controlled, often requiring lipid class switching from pro-inflammatory to anti-inflammatory metabolites.^109^

Pro-inflammatory eicosanoids such as prostaglandin E_2_ (PGE_2_) and leukotriene B_4_ (LTB_4_) derived from AA metabolism via COX and LOX enzymes, respectively, and serve as an early event in the innate inflammatory response, recruiting neutrophils to the injury site.^109^ While this is one example of ‘classical’ pro-inflammatory signalling with AA, important nuances govern inflammatory homeostasis. The same PGE_2_ that recruits neutrophils induces a switch in those neutrophils to stop production of LTB_4_ and begin synthesis of lipoxin A_4_ (LXA_4_) via 15-LOX, which in turn halts neutrophil recruitment and infiltration and stimulates macrophage activity to clean up neutrophil debris (early stages of inflammation resolution).^107,110^ Additionally, low dose aspirin can halt production of PGE_2_ by acetylating COX2 to shift AA conversion to anti-inflammatory lipoxins (also called aspirin-triggered lipoxins, or ATLs).^106^ AA can also be converted into the pro-thrombotic mediator thromboxane A2 via COX enzymes.^112^ EPA and DHA compete with AA for COX binding and production of anti-thrombotic metabolites, such as thromboxane A3 and prostaglandin I3 (PGI3).^113,114^

**Figure 2:**
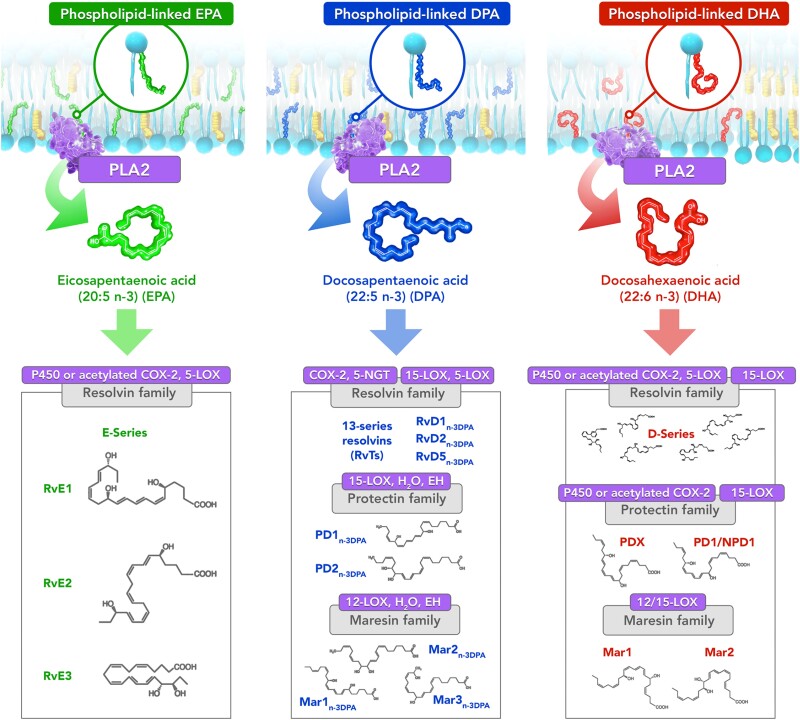
Omega-3 fatty acids EPA, DPA, and DHA give rise to various specialized pro-resolving mediators (SPMs) following release from membrane phospholipids. The O3FAs in the membrane are released enzymatically by PLA_2_ before conversion to bioactive, anti-inflammatory downstream metabolites by P450, 12/15-LOX, COX-2, and acetylated COX-2. These metabolites mediate various anti-inflammatory effects and modulate the transcriptome in resolving inflammation and reducing cytokine activity. 5-NGT, 5-nitrosoglutathione; COX, Cyclooxygenase; EH, epoxide hydrolase; LOX, lipoxygenase; Mar, Maresin; PD, Protectin; PLA_2_, phospholipase A_2_; RvE, E-series resolvins; RvD, D-series resolvins.

EPA, DHA, and DPA give rise to various stereospecific resolvins, maresins, and protectins, each with their own structures and inflammation resolving actions.^107,115,116^ These metabolites elicit their pro-resolving and anti-inflammatory actions by binding various G-protein coupled receptors.^108,117^ While resolvins can derive from each of these O3FAs—E-series resolvins from EPA, D-series resolvins from DHA, and resolvin_n-3DPA_ from DPA—protectins and maresins are only synthesized from DHA or DPA precursors (*Figure 2*). E-series resolvins are generated from a common intermediate, 18-HEPE, which can be produced from EPA by CYP and acetylated COX2. Likewise, all D-series resolvins have a common intermediate upstream of their synthesis, namely 17-hydroxyperoxy-docosahexaenoic acid (17-HpDHA), which is itself produced from DHA metabolism by LOX and acetylated COX2. The SPMs work in concert to stop neutrophil infiltration and initiate phagocytosis of apoptotic debris by macrophages.^106,107^ The balance between acute inflammatory responses and various anti-inflammatory and pro-resolving mediators govern the net consequences of inflammatory conditions.

When unopposed by pro-resolving mediators, the inflammatory response can propagate unimpeded and promote atherosclerotic plaque development in the arterial wall.^118^ Carotid endarterectomy analysis found an inverse relationship between the ratio of resolvin D1-to-LTB_4_ in the plaque and indices of vulnerability.^119^ Similar trends were observed in experimental atherosclerosis using LDL receptor-deficient mice (*Ldlr^−/−^*), and treatment with resolvin D1 significantly improved plaque stability. Another study using a different animal model of atherosclerosis (*ApoE^−/−^*) found an inverse relationship between plaque instability and SPM concentration, specifically resolvin D2 and maresin 1, and a direct correlation with both PGE_2_ and LTB_4_.^120^ Treatment with SPMs once again increased indices of plaque stability, thereby contributing to overall attenuation of atherosclerotic plaque progression. Resolvin E1 treatment of atherosclerotic animals also decreased plaque size and improved features of plaque stability.^121,122^

## 8. EPA mechanisms of action: lipid antioxidant actions

As mentioned earlier, lipids and lipoproteins often contribute substantially to the overall content of atherosclerotic plaques. Under conditions of oxidative stress, lipoproteins such as LDL can become modified, decreasing their affinity for the LDL receptor and possibly increasing their atherogenicity by promoting endothelial dysfunction and macrophage foam cell formation within the atherosclerotic plaque.^63,123,124^ Increased levels of oxidized LDL are associated with acute coronary syndromes and elevated risk for ischaemic events, though the causal role of oxidized LDL in ischaemic events in humans is not well understood.^92,125–127^ In the ANCHOR trial, oxidized LDL levels were reduced with 4 g/day IPE treatment as compared to placebo.^128^  *In vitro* studies of lipoprotein oxidation show the ability of EPA to prevent oxidation of different ApoB and ApoA lipoprotein subfractions, including LDL, small dense LDL (sdLDL), very low dense LDL (VLDL), and HDL, compared with other fatty acids tested including DHA and other TG-lowering agents.^71,129,130^ This finding may result from the particular chemical structure of EPA that allows it to intercalate more effectively into the outer layer of these particles and prevent the propagation of reactive oxygen species (ROS) throughout this layer and the rest of the lipid particle. This effect may result from resonance stabilization of oxygen free radicals arising from the conjugated double bonds along the acyl chain of EPA.^63^

Other studies have shown that upon incorporation into HDL particles, EPA significantly improves its anti-inflammatory properties.^131,132^ Specifically, HDL isolated from patients treated with EPA (as IPE) significantly increased cholesterol efflux from macrophages and decreased vascular cell adhesion molecule-1 (VCAM-1) expression in endothelial cells as compared with HDL isolated from the patients prior to EPA administration.^133^ The same group found similar results in a follow-up study of reconstituted HDL (rHDL), where incorporation of EPA into rHDL particles and subsequent delivery to endothelial cells and macrophages resulted in decreased VCAM-1 expression and increased cholesterol efflux, respectively.^131^

Oxidative damage due to ROS propagation also damages the acyl chains of cell membrane phospholipids, altering distribution of cholesterol, shortening acyl chain, and disrupting integral membrane protein structure and function.^134–137^ As observed in lipoproteins, EPA significantly reduced lipid oxidation in model membrane bilayers exposed to oxidative conditions as compared with other fatty acids and TG-lowering agents.^71,130,138^ In particular, DHA had highly attenuated antioxidant capacity as compared with EPA. This finding may result from distinct membrane orientations of EPA and DHA in membrane bilayers: while EPA adopts an extended, stable conformation within the bilayer, DHA rapidly isomerizes increasing overall membrane fluidity.^139–145^

Under conditions of high glucose, oxidation of membrane lipids occurs more rapidly than that observed with other sugars, leading to cell permeability, cholesterol re-organization into discrete domains, and overall membrane damage.^138,146–149^ Cholesterol domains, which function as nucleating sites for larger extracellular cholesterol crystals, can form following increased cholesterol concentrations in the membrane. These domains can also form from through inhibition of enzymatic cholesterol esterification and oxidative modification of the membrane.^136,150–153^ Inhibition of cholesterol domain formation may attenuate atherosclerosis, as cholesterol crystals can contribute to fibrous cap puncture, NLR family pyrin domain containing 3 (NLRP3) inflammasome activation, cytokine release, inflammatory cell activation and recruitment, eNOS inhibition and oxidative stress following angiotensin II challenge, and tissue injury.^152,154–159^

In model membranes exposed to oxidative stress and high glucose, EPA significantly reduced lipid hydroperoxide and cholesterol domain formation more than TG-lowering agents and other fatty acids.^71,138^ EPA appears to have an optimum combination of acyl chain length and degree of unsaturation to yield maximal antioxidant capacity. Shorter, more saturated O3FAs, such as α-ALA, and eicosatrienoic acid (ETE, 20:3 n-3), and longer O3FAs, such as docosapentaenoic acid (DPA, 22:5 n-3) and DHA, did not inhibit lipid oxidation with the same potency as EPA. Interestingly, DPA inhibited cholesterol domain formation in similar fashion, despite reduced antioxidant capacity, suggesting other mechanisms may also influence domain stability.

## 9. EPA mechanism of action: membrane stabilization

Following oral intake and distribution throughout the body in lipoproteins, lipases liberate EPA and other fatty acids at the target cell surface that then enter the smooth endoplasmic reticulum and undergo esterification at the *sn*-2 position of phospholipids.^160,161^ Within the membrane, O3FAs and O6FAs can modulate the membrane structure and fluidity of surrounding bulk lipids. Recent biophysical analyses of model membranes have shown differences in the structure and fluidity of EPA, DHA, and various O6FAs including arachidonic acid.

As mentioned above, the increased lipid antioxidant capacity of EPA compared with other fatty acids, specifically DHA, results from its favourable membrane orientation. This conclusion is based on studies using small angle X-ray scattering (SAXS) to characterize the structure and relative electron density distribution of membranes prepared in the absence or presence of EPA or DHA.^139,140^ In a recent study, phospholipids containing palmitic acid (16:0) at the *sn*-1 position and EPA (PL-EPA) or DHA (PL-DHA) at the *sn*-2 position were incorporated into model membranes containing physiologic levels of cholesterol and phospholipids.^139^ Membranes enriched with PL-EPA showed relative increases in membrane hydrocarbon core electron density over a broad area from the centre (±0–10 Å), whereas membranes enriched with PL-DHA showed increased electron density near the phospholipid head group region concomitant with disordering in the hydrocarbon core.

These results in membranes indicate that DHA isomerizes more rapidly as compared with EPA when surrounded by bulk phospholipid, causing disruption in van der Waals interactions of surrounding acyl chains. EPA, by contrast, adopted a stable, extended orientation in the membrane in the same plane as the surrounding acyl chains (*Figure 1*). These findings depended highly on the surrounding lipid environment: differences emerged once physiologic levels of phospholipid were added. A previous study showed the same differences in orientation and dynamics for EPA vs. DHA when added to membranes as free fatty acids.^140^ Equimolar combinations of PL-EPA and PL-DHA attenuated the separate effects of each, resulting in membranes with little to no differences in electron density as compared with control membranes. The opposing effects of PL-EPA and PL-DHA on membrane structure and electron density distribution demonstrated experimentally could contribute to the contrasting outcomes of CVOTs of EPA monotherapy compared with EPA/DHA mixed formulations.^62,161^

Due to distinct membrane orientations, EPA and DHA differentially modulate overall membrane fluidity.^141,142,162^ Fluorescence anisotropy techniques showed that EPA treatment lacked significant effect on membrane lipid dynamics at multiple concentrations, while DHA increased fluidity in a concentration-dependent manner.^141^ Membranes in this study were prepared at 50 mol% cholesterol to reproduce physiologic conditions of the plasma membrane. SAXS analysis of these same membrane preparations revealed that EPA maintained normal cholesterol distribution and width at multiple temperatures, while DHA induced cholesterol domain formation.

Recent micropipette aspiration studies in an independent laboratory confirmed these differential effects of EPA and DHA on membrane fluidity and cholesterol distribution.^142^ At high cholesterol levels, EPA maintained uniform cholesterol distribution in model membranes, consistent with the fluorescence anisotropy measurements. DHA, by contrast, redistributed cholesterol into discrete regions and increased fluidity as measured by increased lateral stretching. Other analyses using 2H NMR spectroscopy have shown that PL-DHA undergoes rapid trans-gauche isomerization and preferentially associates with cholesterol-poor regions of the membrane.^163,164^ Another study found that PL-DHA can incorporate into lipid rafts (including cholesterol rich domains) more effectively than PL-EPA, which may lead to disruption of pre-existing domains due to rapid cholesterol redistribution.^144^ Thus it appears that the rapid isomerization of DHA within the membrane lead to increased fluidity and segregation of cholesterol into cholesterol domains as compared with EPA, and this may have downstream effects on integral membrane protein function and signalling.

The membrane-disordering effects of DHA are an oft-studied aspect of its chemistry and may be crucial to its important functions in certain tissues, including the retina and neuronal cell membranes (*Figure 1*). In the retina, rhodopsin must adopt different confirmations on a nanoscale time frame in response to light stimuli, and the increased fluidity caused by DHA may facilitate this function.^165^ DHA-induced membrane lipid rafts may also be crucial for neuronal cell function, as DHA is the most abundant polyunsaturated fatty acid (PUFA) in these cell membranes.^3,166,167^ In atrial cell membranes, increased fluidity has been associated with onset of atrial fibrillation (AF), indicating that O3FAs may be differentially affect the activity of ion channels.^168^ The mechanosensitive cation channel Piezo1, for example, is expressed in cardiomyocytes and translates stretching actions into Ca^2+^ release and ROS production.^169^ This channel is essential to maintaining cardiomyocyte homeostasis and function, as knockout of this protein leads to impaired heart function (decreased ejection fraction and development of cardiomyopathy). Alternatively, overexpression of Piezo1 induces heart failure with reduced ejection fraction and dilated cardiomyopathy concomitant with arrhythmias (specifically ventricular tachycardia, likely due to increased Ca^2+^ release) *in vivo*.^169^ Finally, activation of Piezo1 has been linked to increased AF risk.^170^

Romero *et al.* found differential effects of EPA and DHA on Piezo1 activity in multiple cells types.^171^ Specifically, EPA decreased Piezo1 activity while DHA increased it despite similar decreases in membrane stiffness. They hypothesized that the additional carbon atoms and double bond of DHA further disrupts the lipid environment around Piezo1, leading to such conformational changes that rendered the channel open longer than observed with EPA. Indeed, DHA decreased the melting temperature of treated membranes more than EPA, suggesting incremental increased fluidity with DHA.

In recent clinical trials, treatment with EPA monotherapy as well as with mixed EPA/DHA formulations have been associated with increased rates of AF.^46,60,64,172^ This finding seems to implicate O3FA-mediated AF increases independent of Piezo1. Importantly, EPA monotherapy has been associated with reduced rates of ventricular arrhythmias following successful PCI to treat acute MI.^173^ This result agrees with a secondary analysis of high-risk patients in the Alpha Omega Trial, which found an association between O3FA intake and decreased ventricular arrhythmia-related events and fatal MI.^174^ In another study, Wang *et al.* found that although EPA and DHA both reduced ventricular arrhythmia compared with vehicle control following experimental MI *in vivo*, EPA exhibited superior effects via increased activation of peroxisome proliferator-activated receptor γ (PPARγ) and decreased IL-1β release which in turn decreased synthesis of nerve growth factor (NGF), a key component of sympathetic hyperinnervation and subsequent ventricular arrhythmias.^175^ Also, in REDUCE-IT, IPE treatment significantly reduced cardiac arrest and sudden cardiac death. While this finding may represent an effect on plaque rupture, it might reflect in part a reduction in malignant ventricular arrhythmias. Clearly, more work is needed to characterize more fully the role O3FAs play in AF pathophysiology, and to determine to what extent the differential effects of EPA and DHA on membrane fluidity, structure, and integral membrane protein functions impact CVD outcomes.

## 10. EPA mechanisms of action with: effects on glucose and insulin resistance

**Figure 3:**
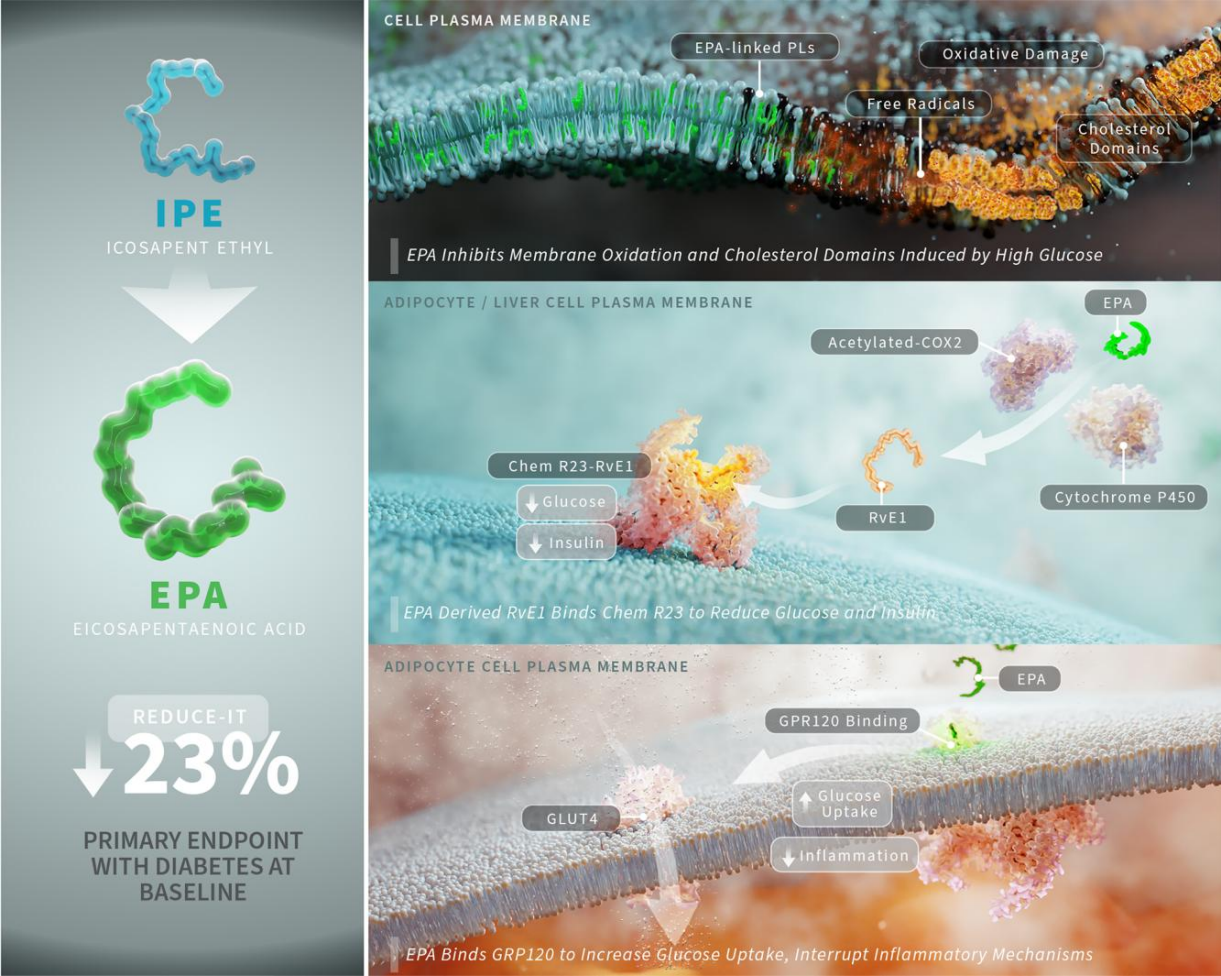
EPA interrupts multiple atherosclerotic and diabetes-related mechanisms caused by glucose. In the subpopulation of REDUCE-IT that had diabetes at baseline, treatment with IPE, which is converted to EPA by lipases in the intestinal lumen, experienced a 23% relative risk reduction in the primary endpoint. Elevated levels of glucose have been shown to increase the rate of oxidation in model membranes and induce formation of cholesterol liquid-crystalline domains. Due to its favourable orientation within the membrane and highly conjugated structure, EPA interrupts free radical propagation and cholesterol domain formation, thereby maintaining cholesterol distribution and preventing further acyl chain degradation. EPA-derived resolvin E1 has also been shown to reduce plasma glucose and insulin levels, a mechanism dependent on its receptor ERV1/ChemR23. Finally, EPA can bind to the free fatty acid receptor GPR120, which can increase GLUT4 translocation to the membrane and increase glucose uptake in adipocytes, as well as interrupt inflammatory signalling to NF-κB. COX2, cyclooxygenase-2; EPA, eicosapentaenoic acid; IPE, icosapent ethyl; RRR, relative risk reduction; RvE1, resolvin E1.

REDUCE-IT randomized many participants with diabetes (59%) or obesity (57%, body mass index ≥ 30).^46^ The risk reduction observed in the overall population was consistent irrespective of diabetes status at baseline (HR with diabetes at baseline = 0.77 (0.68–0.87), *P* < 0.001). Additionally, a sub-analysis of REDUCE-IT showed a significant risk reduction of primary endpoints across tertiles of waist circumference, and the risk reduction was independent of abdominal obesity.^176^ In the subgroup of patients from the JELIS trial diagnosed with diabetes, there was a 22% decrease in incidence of CV events.^177^ Thus, EPA may offer particular benefits for patients with diabetes as compared with EPA/DHA mixed formulations, possibly due to improved insulin sensitivity in addition to the other pleiotropic effects discussed in this review (*Figure 3*).

Insights from several recent animal studies shed light on these differences.^178–180^ In one study, EPA but not DHA, improved glucose tolerance and insulin sensitivity in normal and obese mice fed a high-fat diet enriched in each of the separate O3FAs.^178^ Another study found that EPA, but not an EPA/DHA combination, improved glucose tolerance, reduced fasting blood glucose, improved insulin sensitivity, and reduced weight in mice fed a high-fat diet.^179^ A third study identified one metabolite of EPA, specifically resolvin E1, as playing a key role in improving fasting glucose levels in obese mice through binding the ERV1/ChemR23 receptor.^180^ The immediate precursor to resolvin E1, 18-hydroxyeicosapentaenoic acid (18-HEPE), along with resolvin E1 both fall in liver tissue following high-fat diet intake, and treatment with EPA monotherapy increased concentrations of both eicosanoids. Thus, the benefits of EPA on glucose metabolism may derive from its bioactive metabolites as opposed to direct effects of EPA itself. Because EPA/DHA mixed formulations contain less EPA they may not sufficiently increase EPA levels to generate the eicosanoids that elicit these benefits.

There may also be insulin sensitizing effects mediated through O3FA binding to G-protein coupled receptor 120 (GPR120). Several cell types express this free fatty acid binding protein, including macrophages, adipocytes, intestinal cells, and it can be induced in hepatic Kupffer cells.^181,182^ In the intestine, GPR120 activation can augment secretion of glucagon-like peptide-1 (GLP-1), which has its own beneficial effects on glucose tolerance and is itself mimicked by GLP-1 receptor and mixed incretin agonists.^182–185^ Upon binding free fatty acids, this receptor increases glucose transporter type 4 (GLUT4) translocation and subsequent glucose uptake in adipocytes and increases insulin sensitivity in mice fed O3FA-enriched diets.^181^ This receptor also reduced macrophage activation and inflammatory marker release by activating β-arrestin2 and, in turn, sequestering transforming growth factor-β-activated kinase 1 (TAK1) binding protein-1 (TAB1) away from TAK1 and interrupting the pro-inflammatory signalling cascades initiated by lipopolysaccharide (LPS) or tumour necrosis factor-α (TNF-α). Oh *et al.* elucidated these actions of GPR120 in response to DHA, and these anti-inflammatory effects derive support from subsequent work with EPA showing similar TAK1/TAB1 inhibition in adipocytes following challenge with palmitic acid (16:0) and interruption of inflammatory cascades in adipose tissue from mice fed a high-fat, high-sucrose diet.^186^ Together, the decrease in insulin resistance and inflammatory insult mediated by GPR120 binding O3FAs may help explain the benefits in patients with impaired glucose control.

## 11. EPA mechanisms of action: endothelial functions

Endothelial dysfunction, characterized by a loss of nitric oxide (NO) bioavailability and increased expression of pro-inflammatory adhesion molecules such as VCAM-1 and intercellular adhesion molecule-1 (ICAM-1), is one of the early stages of atherosclerosis and initiators of thrombosis.^187–190^ NO is among the most important signalling molecules in the endothelium, as it regulates vascular tone, inhibits leucocyte adhesion and diapedesis, and interrupts platelet aggregation.^187,191^ The enzyme responsible for NO production in endothelial cells, endothelial nitric oxide synthase (eNOS), is a dimer composed of two identical subunits, each with a reductase and oxygenase domain, connected by a flexible protein strand.^192^ Under normal conditions, eNOS catalyzes the formation of NO via coupling the oxidation of L-arginine with the reduction of molecular oxygen. NO then activates soluble guanylyl cyclase in vascular smooth muscle cells to generate the secondary messenger cyclic guanosine monophosphate (cGMP), which further activates downstream signalling pathways, culminating in vasodilation and reduced inflammation.

**Figure 4:**
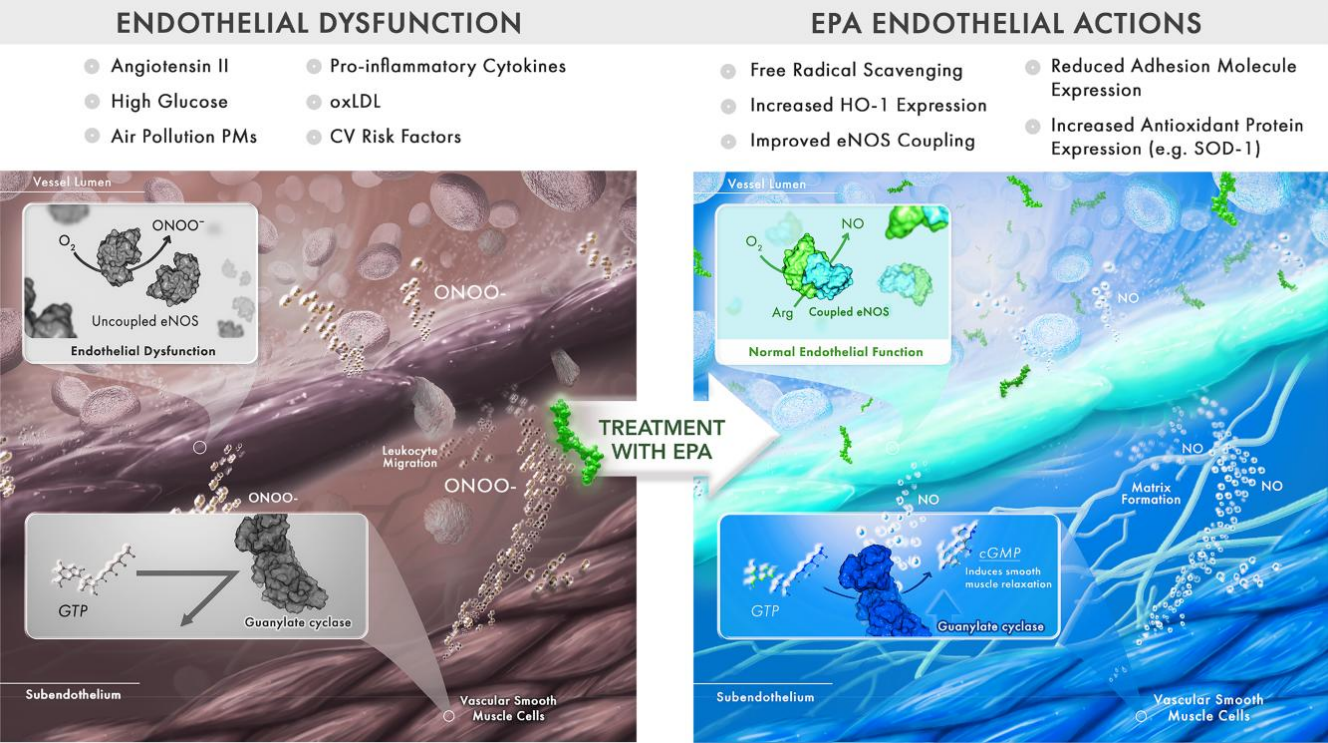
EPA improving endothelial nitric oxide bioavailability depends on eNOS coupling efficiency. Endothelial dysfunction is one of the early stages of atherosclerosis, characterized by the loss of NO bioavailability and adhesion/transendothelial migration (diapedesis) of circulating monocytes. NO is crucial for regulating vascular tone, as it binds gyanalyl guanylyl cyclase in vascular smooth muscle cells which in turn generates cGMP for further downstream signalling pathways leading to vasodilation and reduced inflammatory changes. Under disease conditions, the dimeric eNOS can become uncoupled to favour production of superoxide and peroxynitrite. EPA has been shown to reverse endothelial dysfunction by decreasing adhesion molecule expression, monocyte adhesion, and increasing eNOS coupling efficiency. This constitutes a key atheroprotective mechanism of EPA. Arg, arginine; cGMP, cyclic guanosine monophosphate; CV, cardiovascular; eNOS, endothelial nitric oxide synthase; GTP, guanosine triphosphate; HO-1, heme oxygenase-1; NO, nitric oxide; ONOO−, peroxynitrite; oxLDL, oxidized LDL; PM, particulate matter; SOD-1, superoxide dismutase-1.

However, under disease-like conditions (e.g. high glucose, air pollution, and hypertension) that increase oxidative stress and an abundance of reactive oxygen species, levels of necessary cofactors such as tetrahydrobiopterin (BH_4_) can become insufficient for maintaining the redox reaction above, leading to eNOS ‘uncoupling’ (*Figure 4*).^193–195^ In this state, eNOS favours the production of superoxide (O_2_^−^) rather than NO. O_2_^−^ can then react with NO to form peroxynitrite (ONOO^−^), a cytotoxic radical oxygen species that can oxidize BH_4_ and further promote the loss of this essential cofactor.^196^ The ratio of [NO]/[ONOO^−^] is a key indicator of eNOS coupling efficiency and directly related to the relative amounts of dimeric and monomeric eNOS present in the cell.^197^ Reversing endothelial dysfunction can therefore take many forms, including reducing oxidative stress, increasing NO bioavailability and eNOS coupling, and decreasing pro-inflammatory adhesion molecules and mediators.

*In vitro* studies have shown that treatment with O3FAs can reverse endothelial dysfunction under various disease conditions. In separate studies, either EPA or DHA have decreased expression of VCAM-1 in endothelial cells challenged with LPS and TNF-α separately; these changes correlated with decreased monocyte rolling and adhesion.^198–200^ EPA can also increase eNOS activity by displacing a known inhibitor, caveolin-1, from membrane caveolae.^201^ Using tandem porphyrinic nanosensors, recent studies have shown differential effects of EPA and DHA on eNOS coupling as evidenced by the [NO]/[ONOO^−^] ratio in human umbilical vein endothelial cells (HUVECs) under normal and disease-like conditions.^202,203^ Specifically, EPA increased NO bioavailability concomitant with reduced ONOO^−^ release, leading to an overall 35% increase (*P* < 0.001) in the [NO]/[ONOO^−^] ratio.^202^ This increase was significantly greater than the increase observed with DHA treatment (23%), and AA treatment produced no significant increase.

These changes in eNOS function correlated with modifications in cellular fatty acid levels, specifically the EPA/AA ratio and DPA. EPA and DPA levels increased 10- and 2-fold, respectively, with EPA treatment, and the EPA/AA ratio increased 10-fold. There was no effect on DPA or EPA levels with DHA treatment and only a modest, 2.6-fold increase in the EPA/AA ratio. These changes agree with another study of human THP-1 macrophages, in which EPA but not DHA or AA treatment increased EPA and DPA levels, and EPA treatment also decreased AA levels.^204^ In this study, these changes particular to EPA treatment correlated with increased ABCA1-mediated cholesterol efflux from the macrophages compared with the other FA treatments, which may constitute a novel mechanism of plaque volume reduction with EPA. This trend also prevailed under disease-like conditions.^203^ In this study, sdLDL pre-treated with EPA or DHA was exposed to oxidative conditions and then introduced to endothelial cells. EPA protected against oxidative damage, resulting in increased NO bioavailability compared with DHA. Additionally, the combination of EPA and atorvastatin (as the ortho-hydroxy atorvastatin metabolite) provided incremental increases in the [NO]/[ONOO^−^] ratio in HUVECs challenged with oxidized LDL and high glucose treatment.

As mentioned previously, a sub-study of REDUCE-IT found a significant reduction in MACE with IPE among patients with a history of smoking, indicating that EPA may have beneficial effects in pulmonary tissues or following exposure to air pollution materials.^53^ As much as 20% of CV deaths are related to air pollution exposure, and the endothelium is a key mediator of various ASCVD aetiologies impacted by air pollution.^205^ A recent *in vitro* study showed that, indeed, EPA can also reverse pulmonary endothelial dysfunction and favourably modify protein expression under disease-like conditions with air pollution particulate matter (PM).^206^ In this study, pulmonary ECs were pre-treated with EPA and then challenged with PMs of varying particle size and chemical composition. These PMs significantly reduced the [NO]/[ONOO^−^] ratio, increased expression of soluble ICAM-1, and stimulated expression of cellular proteins and pathways associated with inflammation and oxidative stress. Pre-treatment with EPA increased NO bioavailability without changing eNOS expression, indicating improved eNOS coupling efficiency. EPA also decreased soluble ICAM-1 expression and increased expression of important cytoprotective proteins, such as peroxiredoxin isoforms and heme oxygenase-1 (HO-1). These *in vitro* findings may help explain the observed cardioprotective effects of O3FA intake in small human clinical studies when subjects were exposed to various pollution environments.^207–209^

In addition to its effects on eNOS coupling efficiency, EPA may convey endothelial protection through other mechanisms, including induction of HO-1 expression and activation of the transient receptor potential vanilloid 4 (TRPV4) channel. The cytoprotective actions of HO-1—breaking down heme into biliverdin, carbon monoxide (CO), and free iron—are important for responding to oxidative stress.^210–212^ Both CO and biliverdin possess antioxidant activity, and studies have shown that these products of HO-1 activity can reverse endothelial dysfunction and decrease atherosclerotic lesion size *in vivo*.^213–217^ The specific mechanism of endothelial function improvement appears to involve increased phosphorylation of eNOS at Ser1177 (activating site) by Akt.^213^ Several studies have shown EPA increases HO-1 expression under inflammatory and oxidative challenge by inducing p38 MAPK signalling and increasing nuclear translocation of nuclear factor-erythroid factor 2-related factor 2 (Nrf2), a transcription factor that binds to antioxidant response elements to facilitate expression of HO-1 and other target genes.^206,218,219^

The TRPV4 ion channel is involved in endothelium-dependent vasodilation and NO release.^220^ Recent investigations in genetically modified *Caenorhabditis elegans* and human pulmonary microvascular endothelial cells alike showed that PUFAs, specifically EPA and its cytochrome P450 metabolite 17,18-epoxyeicosatetraenoic acid (17,18-EEQ) are necessary for proper TRPV4 function.^221^ DHA treatment did not produce this effect. The authors hypothesized that part of the activation of TPRV4 observed with 17,18-EEQ is increased membrane fluidity around the channel due to effects of the epoxy residue on the acyl chain—a hypothesis also supported by atomic force microscopy measurements. These data highlight the complex and vital role the surrounding lipid environment plays in channel function, and in this instance, EPA-mediated endothelial function.

## 12. Conclusions

**Figure 5:**
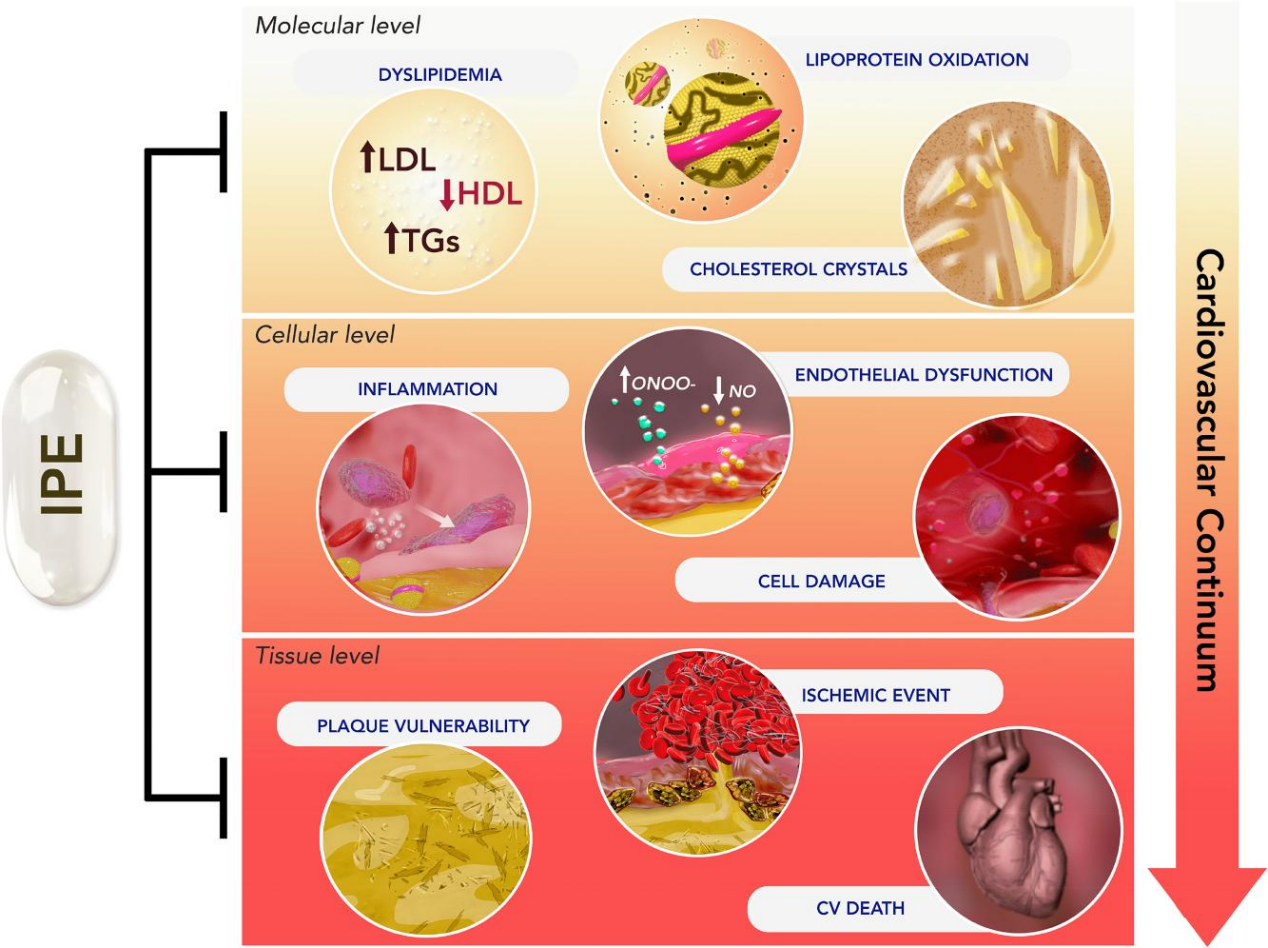
EPA interrupts the cardiovascular disease continuum at multiple points. There are multiple mechanisms associated with the cardiovascular disease continuum, starting with endothelial dysfunction and dyslipidaemia and culminating in ischaemic events, organ damage and death. Clinical trials, most notably REDUCE-IT, showed treatment with icosapent ethyl (IPE) reduced risk of major adverse cardiovascular events by 25%. The active ingredient of IPE, EPA, has shown beneficial activity at several points along the continuum, all of which contribute to the overall risk reduction.

Elucidating the roles that O3FAs may have in the treatment of CVD has proved elusive and often contradictory. Diets rich in O3FAs are associated with reduced incidence of CVD events, but treatment with O3FA supplements containing EPA and DHA for primary and secondary prevention have not shown consistent benefit. Pharmaceutical-grade, EPA-only preparations (i.e. IPE) have however shown CV event reduction as observed in JELIS and REDUCE-IT. This benefit has not been replicated in pharmaceutical-grade, mixed EPA/DHA preparations at comparable doses (OMEMI, STRENGTH) nor predicted by traditional lipid and inflammatory biomarkers. These differences in clinical benefit for CV patients indicate selective mechanisms of action with EPA compared with DHA beyond TG lowering, through which EPA can interrupt the continuum of CVD at multiple points (*Figure 5*). These mechanisms include distinct effects on lipid oxidation, endothelial function, inflammation, membrane structure and fluidity, and plaque stability (*Table 2*). Together, these data indicate particular benefits for patients at risk for CVD that are not shared by formulations containing DHA, including FODS and pharmaceutical preparations. Characterizing the multifactorial mechanisms of action of EPA beyond TG lowering will lead to further insights into atherosclerosis and potential strategies for treatment.

## Data Availability

No new data were generated or analysed in support of this manuscript.
